# Methyl 9-(4-bromo­phen­yl)-8a,9,9a,10,11,12,13,14a-octa­hydro-8*H*-benzo[*f*]chromeno[3,4-*b*]indolizine-8a-car­box­ylate

**DOI:** 10.1107/S1600536809037994

**Published:** 2009-09-26

**Authors:** B. Gunasekaran, S. Kathiravan, R. Raghunathan, G. Chakkaravarthi, V. Manivannan

**Affiliations:** a Department of Physics, AMET University, Kanathur, Chennai 603 112, India; b Department of Organic Chemistry, University of Madras, Guindy Campus, Chennai 600 025, India; c Department of Physics, CPCL Polytechnic College, Chennai 600 068, India; d Department of Research and Development, PRIST University, Vallam, Thanjavur 613 403, Tamil Nadu, India

## Abstract

In the title compound, C_27_H_26_BrNO_3_, the mean plane of the naphthalene ring system makes a dihedral angle of 22.0 (1)° with the bromo-substituted benzene ring. The pyrrolidine and piperidine rings exhibit envelope and chair conformations, respectively. An inter­molecular C—H⋯Br inter­action is observed.

## Related literature

For the biological activity of indolizine derivatives, see: Gundersen *et al.* (2003 ▶); Teklu *et al.* (2005 ▶); Foster *et al.* (1995 ▶); Malonne *et al.* (1998 ▶); Medda *et al.* (2003 ▶). For related structures, see: Gunasekaran *et al.* (2009 ▶); Kamala *et al.* (2009 ▶).
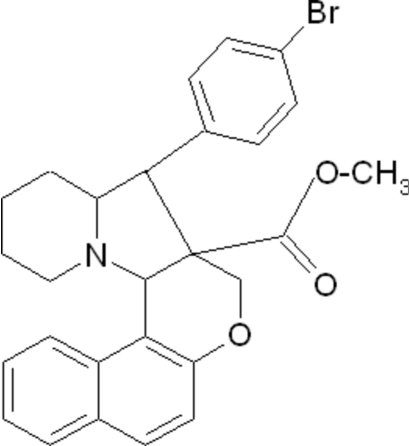


## Experimental

### 

#### Crystal data

C_27_H_26_BrNO_3_
*M* *_r_* = 492.40
Trigonal, 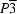

*a* = 18.4405 (8) Å
*c* = 11.4828 (8) Å
*V* = 3381.6 (3) Å^3^
*Z* = 6
Mo *K*α radiation
μ = 1.85 mm^−1^
*T* = 293 K
0.25 × 0.20 × 0.20 mm

#### Data collection

Bruker Kappa APEXII CCD diffractometer
Absorption correction: multi-scan (**SADABS**; Sheldrick, 1996 ▶) *T*_min_ = 0.654, *T*_max_ = 0.708
23085 measured reflections
5338 independent reflections
2509 reflections with *I* > 2σ(*I*)
*R*_int_ = 0.113

#### Refinement

*R*[*F* ^2^ > 2σ(*F* ^2^)] = 0.042
*wR*(*F* ^2^) = 0.108
*S* = 0.85
5338 reflections
290 parameters
2 restraints
H-atom parameters constrained
Δρ_max_ = 0.85 e Å^−3^
Δρ_min_ = −0.33 e Å^−3^

### 

Data collection: *APEX2* (Bruker, 2004 ▶); cell refinement: *SAINT* (Bruker, 2004 ▶); data reduction: *SAINT*; program(s) used to solve structure: *SHELXS97* (Sheldrick, 2008 ▶); program(s) used to refine structure: *SHELXL97* (Sheldrick, 2008 ▶); molecular graphics: *PLATON* (Spek, 2009 ▶); software used to prepare material for publication: *SHELXL97*.

## Supplementary Material

Crystal structure: contains datablocks global, I. DOI: 10.1107/S1600536809037994/is2463sup1.cif

Structure factors: contains datablocks I. DOI: 10.1107/S1600536809037994/is2463Isup2.hkl

Additional supplementary materials:  crystallographic information; 3D view; checkCIF report

## Figures and Tables

**Table 1:** Hydrogen-bond geometry (Å, °)

*D*—H⋯*A*	*D*—H	H⋯*A*	*D*⋯*A*	*D*—H⋯*A*
C15—H15*B*⋯Br1^i^	0.97	2.73	3.588 (3)	147

Symmetry code: (i) 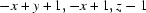.

**Fig. 1.**
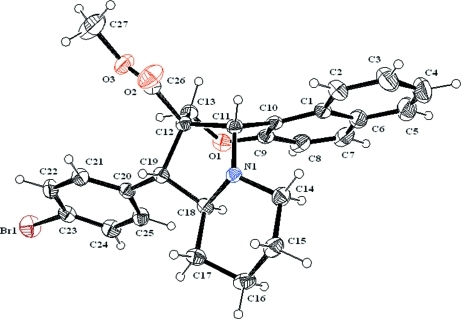
The molecular structure of the title compound, with atom labels and 30% probability displacement ellipsoids for non-H atoms.

## supplementary crystallographic information

### Comment

Indolizine derivatives exhibit antioxidative (Teklu *et al.*, 2005), antiherpes (Foster *et al.*, 1995), anti-inflammatory (Malonne *et al.*, 1998) and antiviral (Medda *et al.*, 2003) activities. In addition, indolizines are used as antimycobacterial agents against mycobacterial tuberculosis (Gundersen *et al.*, 2003).

The geometric parameters of the title compound (Fig. 1) agree well with reported similar structures (Gunasekaran *et al.*, 2009; Kamala *et al.*, 2009). The mean plane of naphthalene ring makes the dihedral angle of 22.0 (1)° with the benzene ring. The pyrrolidine ring exhibits an envelope conformation and the piperidine (N1/C14–C18) ring exhibits a chair conformation [C16—C15—C14—N1 = 56.3 (3)° and C16—C17—C18—N1 = -53.9 (3)°]. The sum of bond angles around N1 [338.3 (2)°] indicates *sp^3^* hybridization.

### Experimental

A mixture of (*Z*)-methyl 2-((1-formylnaphthalen-2-yloxy)methyl) -3-(4-bromophenyl)acrylate (20 mmol) and piperidine-2-carboxylic acid (30 mmol) were refluxed in benzene for 20 h and the solvent was removed under reduced pressure. The crude product was subjected to column chromatography to get the pure product. Chloroform and methanol (1:1) solvent mixture was used for the crystallization under slow evaporation method.

### Refinement

H atoms were positioned geometrically and refined using riding model with C—H = 0.93 Å and *U*_iso_(H) = 1.2*U*_eq_(C) for aromatic C—H, C—H = 0.98 Å and *U*_iso_(H) = 1.2*U*_eq_(C) for CH, C—H = 0.97 Å and *U*_iso_(H) = 1.2*U*_eq_(C) for CH_2_, and C—H = 0.96 Å and *U*_iso_(H) = 1.5*U*_eq_(C) for CH_3_. The components of the anisotropic displacement parameters in direction of the bond of C1, C2, C26 and O2 were restrained to be equal within an effective standard deviation of 0.001 using the DELU command in *SHELXL* (Sheldrick, 2008).

### Crystal data

**Table:**

C_27_H_26_BrNO_3_	*D*_x_ = 1.451 Mg m^−^^3^
*M**_r_* = 492.40	Mo *K*α radiation, λ = 0.71073 Å
Trigonal, *P*3	Cell parameters from 4759 reflections
Hall symbol: -P 3	θ = 2.2–25.7°
*a* = 18.4405 (8) Å	µ = 1.85 mm^−^^1^
*c* = 11.4828 (8) Å	*T* = 293 K
*V* = 3381.6 (3) Å^3^	Block, colourless
*Z* = 6	0.25 × 0.20 × 0.20 mm
*F*(000) = 1524	

### Data collection

**Table:**

Bruker Kappa APEXII CCD diffractometer	5338 independent reflections
Radiation source: fine-focus sealed tube	2509 reflections with *I* > 2σ(*I*)
graphite	*R*_int_ = 0.113
Detector resolution: 0 pixels mm^-1^	θ_max_ = 27.8°, θ_min_ = 2.2°
ω and φ scans	*h* = −22→24
Absorption correction: multi-scan (*SADABS*; Sheldrick, 1996)	*k* = −24→24
*T*_min_ = 0.654, *T*_max_ = 0.708	*l* = −8→15
23085 measured reflections	

### Refinement

**Table:**

Refinement on *F*^2^	Primary atom site location: structure-invariant direct methods
Least-squares matrix: full	Secondary atom site location: difference Fourier map
*R*[*F*^2^ > 2σ(*F*^2^)] = 0.042	Hydrogen site location: inferred from neighbouring sites
*wR*(*F*^2^) = 0.108	H-atom parameters constrained
*S* = 0.85	*w* = 1/[σ^2^(*F*_o_^2^) + (0.0452*P*)^2^] where *P* = (*F*_o_^2^ + 2*F*_c_^2^)/3
5338 reflections	(Δ/σ)_max_ < 0.001
290 parameters	Δρ_max_ = 0.85 e Å^−^^3^
2 restraints	Δρ_min_ = −0.33 e Å^−^^3^

### Fractional atomic coordinates and isotropic or equivalent isotropic displacement parameters (Å^2^)

**Table:**

	*x*	*y*	*z*	*U*_iso_*/*U*_eq_	
C1	0.32587 (18)	0.03952 (17)	−0.2836 (3)	0.0450 (7)	
C2	0.2679 (2)	0.0537 (2)	−0.3441 (3)	0.0616 (9)	
H2	0.2401	0.0769	−0.3050	0.074*	
C3	0.2515 (3)	0.0339 (2)	−0.4604 (3)	0.0850 (13)	
H3	0.2123	0.0432	−0.4982	0.102*	
C4	0.2927 (3)	0.0004 (2)	−0.5221 (3)	0.0859 (13)	
H4	0.2821	−0.0115	−0.6011	0.103*	
C5	0.3479 (3)	−0.0149 (2)	−0.4667 (3)	0.0786 (12)	
H5	0.3752	−0.0375	−0.5082	0.094*	
C6	0.3654 (2)	0.00269 (18)	−0.3465 (3)	0.0564 (9)	
C7	0.4228 (2)	−0.01415 (18)	−0.2873 (3)	0.0627 (10)	
H7	0.4519	−0.0349	−0.3286	0.075*	
C8	0.43567 (19)	−0.00053 (17)	−0.1725 (3)	0.0513 (8)	
H8	0.4704	−0.0156	−0.1336	0.062*	
C9	0.39688 (17)	0.03636 (16)	−0.1111 (2)	0.0384 (7)	
C10	0.34630 (16)	0.06057 (15)	−0.1639 (2)	0.0356 (7)	
C11	0.32309 (16)	0.11839 (16)	−0.1002 (2)	0.0341 (6)	
H11	0.2669	0.1056	−0.1242	0.041*	
C12	0.32533 (15)	0.11112 (16)	0.0348 (2)	0.0333 (6)	
C13	0.34153 (18)	0.04079 (17)	0.0692 (2)	0.0431 (7)	
H13A	0.3526	0.0438	0.1522	0.052*	
H13B	0.2920	−0.0127	0.0531	0.052*	
C14	0.41242 (18)	0.23382 (17)	−0.2399 (2)	0.0434 (7)	
H14A	0.3650	0.2180	−0.2912	0.052*	
H14B	0.4416	0.2053	−0.2663	0.052*	
C15	0.47016 (18)	0.32660 (18)	−0.2460 (2)	0.0477 (8)	
H15A	0.4395	0.3550	−0.2268	0.057*	
H15B	0.4914	0.3422	−0.3247	0.057*	
C16	0.54293 (18)	0.35396 (18)	−0.1621 (3)	0.0492 (8)	
H16A	0.5765	0.3293	−0.1850	0.059*	
H16B	0.5782	0.4144	−0.1644	0.059*	
C17	0.50984 (18)	0.32634 (16)	−0.0398 (2)	0.0425 (7)	
H17A	0.5562	0.3404	0.0128	0.051*	
H17B	0.4815	0.3558	−0.0140	0.051*	
C18	0.44950 (16)	0.23290 (15)	−0.0361 (2)	0.0329 (6)	
H18	0.4799	0.2029	−0.0508	0.039*	
C19	0.39796 (15)	0.19968 (16)	0.0742 (2)	0.0323 (6)	
H19	0.3722	0.2342	0.0882	0.039*	
C20	0.44263 (16)	0.20142 (16)	0.1853 (2)	0.0326 (6)	
C21	0.40965 (17)	0.20588 (17)	0.2912 (2)	0.0395 (7)	
H21	0.3612	0.2096	0.2925	0.047*	
C22	0.44673 (19)	0.20491 (18)	0.3956 (2)	0.0454 (8)	
H22	0.4232	0.2072	0.4662	0.054*	
C23	0.51857 (18)	0.20047 (17)	0.3933 (2)	0.0414 (7)	
C24	0.55532 (18)	0.19869 (17)	0.2895 (3)	0.0461 (8)	
H24	0.6053	0.1979	0.2888	0.055*	
C25	0.51612 (17)	0.19814 (17)	0.1866 (2)	0.0426 (7)	
H25	0.5397	0.1955	0.1162	0.051*	
C26	0.24417 (18)	0.09807 (19)	0.0883 (3)	0.0456 (7)	
C27	0.1435 (2)	0.0356 (3)	0.2332 (4)	0.0985 (14)	
H27A	0.1533	0.0860	0.2714	0.148*	
H27B	0.1240	−0.0091	0.2891	0.148*	
H27C	0.1021	0.0211	0.1735	0.148*	
N1	0.38286 (13)	0.20806 (12)	−0.12185 (17)	0.0301 (5)	
O1	0.41141 (11)	0.04609 (11)	0.00670 (17)	0.0446 (5)	
O2	0.20524 (13)	0.12926 (15)	0.0531 (2)	0.0685 (7)	
O3	0.22148 (12)	0.04877 (13)	0.18093 (17)	0.0569 (6)	
Br1	0.56763 (2)	0.19502 (2)	0.53512 (3)	0.06620 (16)	

### Atomic displacement parameters (Å^2^)

**Table:**

	*U*^11^	*U*^22^	*U*^33^	*U*^12^	*U*^13^	*U*^23^
C1	0.0421 (18)	0.0299 (16)	0.0472 (19)	0.0063 (14)	0.0072 (13)	−0.0069 (14)
C2	0.056 (2)	0.060 (2)	0.052 (2)	0.0171 (17)	−0.0091 (16)	−0.0117 (17)
C3	0.091 (3)	0.073 (3)	0.059 (3)	0.016 (2)	−0.019 (2)	−0.012 (2)
C4	0.116 (4)	0.060 (3)	0.052 (3)	0.021 (3)	0.002 (3)	−0.016 (2)
C5	0.110 (3)	0.042 (2)	0.061 (3)	0.022 (2)	0.020 (2)	−0.0113 (19)
C6	0.070 (2)	0.0282 (18)	0.053 (2)	0.0107 (17)	0.0124 (18)	−0.0059 (15)
C7	0.074 (3)	0.0336 (19)	0.077 (3)	0.0245 (19)	0.036 (2)	0.0021 (18)
C8	0.056 (2)	0.0335 (18)	0.068 (2)	0.0251 (16)	0.0165 (18)	0.0085 (16)
C9	0.0382 (17)	0.0246 (15)	0.0459 (19)	0.0109 (14)	0.0076 (14)	0.0020 (13)
C10	0.0324 (16)	0.0240 (15)	0.0413 (17)	0.0071 (13)	0.0047 (13)	−0.0016 (12)
C11	0.0281 (15)	0.0342 (16)	0.0382 (16)	0.0142 (13)	−0.0002 (12)	−0.0010 (13)
C12	0.0313 (16)	0.0295 (15)	0.0368 (16)	0.0134 (13)	0.0027 (12)	0.0023 (12)
C13	0.0474 (19)	0.0340 (17)	0.0440 (17)	0.0175 (15)	0.0040 (14)	0.0025 (13)
C14	0.0450 (18)	0.0462 (19)	0.0402 (17)	0.0236 (16)	0.0022 (14)	0.0055 (14)
C15	0.051 (2)	0.046 (2)	0.0464 (18)	0.0243 (16)	0.0104 (15)	0.0115 (15)
C16	0.0425 (18)	0.0320 (17)	0.069 (2)	0.0157 (15)	0.0108 (16)	0.0068 (15)
C17	0.0395 (18)	0.0322 (16)	0.0538 (19)	0.0165 (14)	−0.0031 (14)	−0.0018 (14)
C18	0.0354 (16)	0.0291 (15)	0.0369 (15)	0.0182 (13)	−0.0024 (13)	−0.0020 (12)
C19	0.0321 (16)	0.0315 (15)	0.0383 (16)	0.0197 (13)	−0.0019 (12)	−0.0035 (12)
C20	0.0346 (16)	0.0288 (15)	0.0370 (16)	0.0178 (13)	−0.0026 (12)	−0.0039 (12)
C21	0.0344 (16)	0.0452 (18)	0.0452 (18)	0.0246 (15)	−0.0037 (14)	−0.0045 (14)
C22	0.050 (2)	0.055 (2)	0.0356 (17)	0.0298 (17)	−0.0023 (14)	−0.0059 (14)
C23	0.0450 (19)	0.0389 (18)	0.0412 (18)	0.0217 (15)	−0.0112 (14)	0.0008 (13)
C24	0.0398 (18)	0.0508 (19)	0.056 (2)	0.0286 (16)	−0.0092 (15)	−0.0005 (15)
C25	0.0427 (18)	0.0492 (19)	0.0432 (18)	0.0285 (15)	−0.0001 (14)	−0.0020 (14)
C26	0.0377 (18)	0.0465 (19)	0.0462 (19)	0.0162 (14)	0.0011 (14)	−0.0031 (15)
C27	0.079 (3)	0.108 (3)	0.103 (3)	0.043 (3)	0.044 (2)	0.021 (3)
N1	0.0292 (12)	0.0280 (12)	0.0332 (12)	0.0143 (10)	0.0022 (10)	0.0007 (10)
O1	0.0462 (13)	0.0353 (12)	0.0571 (14)	0.0238 (10)	−0.0026 (10)	−0.0004 (10)
O2	0.0512 (15)	0.0940 (19)	0.0727 (16)	0.0455 (13)	0.0179 (12)	0.0238 (13)
O3	0.0493 (14)	0.0684 (15)	0.0484 (13)	0.0260 (12)	0.0151 (10)	0.0127 (11)
Br1	0.0694 (3)	0.0800 (3)	0.0538 (2)	0.0408 (2)	−0.01750 (18)	0.00658 (18)

### Geometric parameters (Å, °)

**Table:**

C1—C2	1.406 (4)	C15—C16	1.518 (4)
C1—C6	1.417 (4)	C15—H15A	0.9700
C1—C10	1.427 (4)	C15—H15B	0.9700
C2—C3	1.377 (4)	C16—C17	1.515 (4)
C2—H2	0.9300	C16—H16A	0.9700
C3—C4	1.390 (6)	C16—H16B	0.9700
C3—H3	0.9300	C17—C18	1.514 (4)
C4—C5	1.344 (5)	C17—H17A	0.9700
C4—H4	0.9300	C17—H17B	0.9700
C5—C6	1.418 (5)	C18—N1	1.459 (3)
C5—H5	0.9300	C18—C19	1.516 (3)
C6—C7	1.416 (5)	C18—H18	0.9800
C7—C8	1.341 (4)	C19—C20	1.511 (3)
C7—H7	0.9300	C19—H19	0.9800
C8—C9	1.399 (4)	C20—C21	1.380 (3)
C8—H8	0.9300	C20—C25	1.387 (4)
C9—C10	1.361 (4)	C21—C22	1.385 (4)
C9—O1	1.374 (3)	C21—H21	0.9300
C10—C11	1.521 (4)	C22—C23	1.368 (4)
C11—N1	1.479 (3)	C22—H22	0.9300
C11—C12	1.558 (3)	C23—C24	1.379 (4)
C11—H11	0.9800	C23—Br1	1.890 (3)
C12—C26	1.521 (4)	C24—C25	1.383 (4)
C12—C13	1.521 (4)	C24—H24	0.9300
C12—C19	1.574 (3)	C25—H25	0.9300
C13—O1	1.435 (3)	C26—O2	1.193 (3)
C13—H13A	0.9700	C26—O3	1.324 (3)
C13—H13B	0.9700	C27—O3	1.461 (4)
C14—N1	1.450 (3)	C27—H27A	0.9600
C14—C15	1.498 (4)	C27—H27B	0.9600
C14—H14A	0.9700	C27—H27C	0.9600
C14—H14B	0.9700		
			
C2—C1—C6	117.4 (3)	C16—C15—H15B	109.5
C2—C1—C10	123.2 (3)	H15A—C15—H15B	108.1
C6—C1—C10	119.4 (3)	C17—C16—C15	109.6 (2)
C3—C2—C1	121.0 (3)	C17—C16—H16A	109.7
C3—C2—H2	119.5	C15—C16—H16A	109.7
C1—C2—H2	119.5	C17—C16—H16B	109.7
C2—C3—C4	121.1 (4)	C15—C16—H16B	109.7
C2—C3—H3	119.4	H16A—C16—H16B	108.2
C4—C3—H3	119.4	C18—C17—C16	110.6 (2)
C5—C4—C3	119.4 (4)	C18—C17—H17A	109.5
C5—C4—H4	120.3	C16—C17—H17A	109.5
C3—C4—H4	120.3	C18—C17—H17B	109.5
C4—C5—C6	121.5 (4)	C16—C17—H17B	109.5
C4—C5—H5	119.2	H17A—C17—H17B	108.1
C6—C5—H5	119.2	N1—C18—C17	111.3 (2)
C7—C6—C1	118.7 (3)	N1—C18—C19	100.2 (2)
C7—C6—C5	121.8 (3)	C17—C18—C19	116.0 (2)
C1—C6—C5	119.5 (4)	N1—C18—H18	109.6
C8—C7—C6	120.8 (3)	C17—C18—H18	109.6
C8—C7—H7	119.6	C19—C18—H18	109.6
C6—C7—H7	119.6	C20—C19—C18	118.2 (2)
C7—C8—C9	120.0 (3)	C20—C19—C12	115.9 (2)
C7—C8—H8	120.0	C18—C19—C12	102.47 (19)
C9—C8—H8	120.0	C20—C19—H19	106.5
C10—C9—O1	121.1 (2)	C18—C19—H19	106.5
C10—C9—C8	122.4 (3)	C12—C19—H19	106.5
O1—C9—C8	116.5 (3)	C21—C20—C25	117.6 (2)
C9—C10—C1	118.1 (3)	C21—C20—C19	119.6 (2)
C9—C10—C11	119.7 (2)	C25—C20—C19	122.8 (2)
C1—C10—C11	121.7 (3)	C20—C21—C22	121.8 (3)
N1—C11—C10	112.9 (2)	C20—C21—H21	119.1
N1—C11—C12	103.10 (19)	C22—C21—H21	119.1
C10—C11—C12	112.9 (2)	C23—C22—C21	118.9 (3)
N1—C11—H11	109.2	C23—C22—H22	120.6
C10—C11—H11	109.2	C21—C22—H22	120.6
C12—C11—H11	109.2	C22—C23—C24	121.4 (2)
C26—C12—C13	111.0 (2)	C22—C23—Br1	119.3 (2)
C26—C12—C11	110.4 (2)	C24—C23—Br1	119.3 (2)
C13—C12—C11	110.9 (2)	C23—C24—C25	118.5 (3)
C26—C12—C19	107.9 (2)	C23—C24—H24	120.7
C13—C12—C19	112.5 (2)	C25—C24—H24	120.7
C11—C12—C19	103.98 (19)	C24—C25—C20	121.8 (3)
O1—C13—C12	111.4 (2)	C24—C25—H25	119.1
O1—C13—H13A	109.3	C20—C25—H25	119.1
C12—C13—H13A	109.3	O2—C26—O3	122.9 (3)
O1—C13—H13B	109.3	O2—C26—C12	124.7 (3)
C12—C13—H13B	109.3	O3—C26—C12	112.4 (3)
H13A—C13—H13B	108.0	O3—C27—H27A	109.5
N1—C14—C15	110.7 (2)	O3—C27—H27B	109.5
N1—C14—H14A	109.5	H27A—C27—H27B	109.5
C15—C14—H14A	109.5	O3—C27—H27C	109.5
N1—C14—H14B	109.5	H27A—C27—H27C	109.5
C15—C14—H14B	109.5	H27B—C27—H27C	109.5
H14A—C14—H14B	108.1	C14—N1—C18	114.1 (2)
C14—C15—C16	110.8 (2)	C14—N1—C11	118.1 (2)
C14—C15—H15A	109.5	C18—N1—C11	106.10 (18)
C16—C15—H15A	109.5	C9—O1—C13	112.0 (2)
C14—C15—H15B	109.5	C26—O3—C27	113.0 (3)
			
C6—C1—C2—C3	−1.2 (5)	N1—C18—C19—C12	42.9 (2)
C10—C1—C2—C3	178.6 (3)	C17—C18—C19—C12	162.8 (2)
C1—C2—C3—C4	−0.9 (6)	C26—C12—C19—C20	90.7 (3)
C2—C3—C4—C5	1.6 (6)	C13—C12—C19—C20	−32.0 (3)
C3—C4—C5—C6	−0.1 (6)	C11—C12—C19—C20	−152.0 (2)
C2—C1—C6—C7	−178.6 (3)	C26—C12—C19—C18	−139.1 (2)
C10—C1—C6—C7	1.6 (4)	C13—C12—C19—C18	98.1 (2)
C2—C1—C6—C5	2.5 (4)	C11—C12—C19—C18	−21.9 (2)
C10—C1—C6—C5	−177.3 (3)	C18—C19—C20—C21	153.2 (2)
C4—C5—C6—C7	179.2 (3)	C12—C19—C20—C21	−84.7 (3)
C4—C5—C6—C1	−1.9 (5)	C18—C19—C20—C25	−27.3 (4)
C1—C6—C7—C8	4.1 (5)	C12—C19—C20—C25	94.9 (3)
C5—C6—C7—C8	−177.0 (3)	C25—C20—C21—C22	−1.7 (4)
C6—C7—C8—C9	−4.6 (5)	C19—C20—C21—C22	177.9 (3)
C7—C8—C9—C10	−0.8 (4)	C20—C21—C22—C23	0.8 (4)
C7—C8—C9—O1	178.3 (3)	C21—C22—C23—C24	1.3 (4)
O1—C9—C10—C1	−172.6 (2)	C21—C22—C23—Br1	−177.7 (2)
C8—C9—C10—C1	6.5 (4)	C22—C23—C24—C25	−2.5 (4)
O1—C9—C10—C11	15.6 (4)	Br1—C23—C24—C25	176.5 (2)
C8—C9—C10—C11	−165.3 (2)	C23—C24—C25—C20	1.6 (4)
C2—C1—C10—C9	173.5 (3)	C21—C20—C25—C24	0.4 (4)
C6—C1—C10—C9	−6.7 (4)	C19—C20—C25—C24	−179.1 (2)
C2—C1—C10—C11	−14.9 (4)	C13—C12—C26—O2	−159.1 (3)
C6—C1—C10—C11	164.9 (3)	C11—C12—C26—O2	−35.8 (4)
C9—C10—C11—N1	89.9 (3)	C19—C12—C26—O2	77.2 (3)
C1—C10—C11—N1	−81.6 (3)	C13—C12—C26—O3	22.0 (3)
C9—C10—C11—C12	−26.5 (3)	C11—C12—C26—O3	145.3 (2)
C1—C10—C11—C12	162.0 (2)	C19—C12—C26—O3	−101.6 (3)
N1—C11—C12—C26	108.5 (2)	C15—C14—N1—C18	−55.9 (3)
C10—C11—C12—C26	−129.3 (2)	C15—C14—N1—C11	178.3 (2)
N1—C11—C12—C13	−128.1 (2)	C17—C18—N1—C14	54.8 (3)
C10—C11—C12—C13	−6.0 (3)	C19—C18—N1—C14	178.1 (2)
N1—C11—C12—C19	−7.0 (2)	C17—C18—N1—C11	−173.3 (2)
C10—C11—C12—C19	115.1 (2)	C19—C18—N1—C11	−50.0 (2)
C26—C12—C13—O1	172.0 (2)	C10—C11—N1—C14	42.9 (3)
C11—C12—C13—O1	48.9 (3)	C12—C11—N1—C14	165.0 (2)
C19—C12—C13—O1	−67.0 (3)	C10—C11—N1—C18	−86.7 (2)
N1—C14—C15—C16	56.3 (3)	C12—C11—N1—C18	35.5 (2)
C14—C15—C16—C17	−56.7 (3)	C10—C9—O1—C13	30.1 (3)
C15—C16—C17—C18	55.2 (3)	C8—C9—O1—C13	−149.1 (2)
C16—C17—C18—N1	−53.9 (3)	C12—C13—O1—C9	−63.2 (3)
C16—C17—C18—C19	−167.6 (2)	O2—C26—O3—C27	1.2 (4)
N1—C18—C19—C20	171.6 (2)	C12—C26—O3—C27	−180.0 (3)
C17—C18—C19—C20	−68.5 (3)		

### Hydrogen-bond geometry (Å, °)

**Table:**

*D*—H···*A*	*D*—H	H···*A*	*D*···*A*	*D*—H···*A*
C15—H15B···Br1^i^	0.97	2.73	3.588 (3)	147

Symmetry codes: (i) −*x*+*y*+1, −*x*+1, *z*−1.
